# Which Environmental Factors Have the Highest Impact on the Performance of People Experiencing Difficulties in Capacity?

**DOI:** 10.3390/ijerph13040416

**Published:** 2016-04-12

**Authors:** Verena Loidl, Cornelia Oberhauser, Carolina Ballert, Michaela Coenen, Alarcos Cieza, Carla Sabariego

**Affiliations:** 1 Department of Medical Informatics, Biometry and Epidemiology–IBE, Ludwig-Maximilians-University (LMU), Munich 81377, Germany; Loidl_Veren@web.de (V.L.); cornelia.oberhauser@med.lmu.de (C.O.); michaela.coenen@med.lmu.de (M.C.); 2 Swiss Paraplegic Research, Nottwil 6207, Switzerland; carolina.ballert@paraplegie.ch; 3 Blindness and Deafness Prevention, Disability and Rehabilitation (BDD), World Health Organization, Geneva 1211, Switzerland; acieza@who.int

**Keywords:** environmental factors, international classification of functioning, disability and health, disability evaluation (MeSH), data collection (MeSH), health surveys (MeSH), capacity, performance, random forest

## Abstract

Disability is understood by the World Health Organization (WHO) as the outcome of the interaction between a health condition and personal and environmental factors. Comprehensive data about environmental factors is therefore essential to understand and influence disability. We aimed to identify which environmental factors have the highest impact on the performance of people with mild, moderate and severe difficulties in capacity, who are at risk of experiencing disability to different extents, using data from a pilot study of the WHO Model Disability Survey in Cambodia and random forest regression. Hindering or facilitating aspects of places to socialize in community activities, transportation and natural environment as well as use and need of personal assistance and use of medication on a regular basis were the most important environmental factors across groups. Hindering or facilitating aspects of the general environment were the most relevant in persons experiencing mild levels of difficulties in capacity, while social support, attitudes of others and use of medication on a regular basis were highly relevant for the performance of persons experiencing moderate to higher levels of difficulties in capacity. Additionally, we corroborate the high importance of the use and need of assistive devices for people with severe difficulties in capacity.

## 1. Introduction

Disability is understood by the World Health Organization (WHO) as the negative outcome of the interaction between a health condition and personal and environmental factors (EFs). This understanding of disability—an umbrella term for impairments, activity limitations and participation restrictions—is based on the biopsychosocial model proposed by WHO in the International Classification of Functioning, Disability and Health (ICF) [1]. According to WHO, 15% of the world’s population have significant disability and the number of people with disabilities is growing, due to populations aging and increase of chronic health conditions with associated disability [2]. 

In the ICF model, disability is understood as a universal experience on a continuum ranging from no to complete levels of disability [1]. The level of disability on this continuum is not static, but can considerably change depending on the hindering or facilitating impact of EFs. Environmental factors are broadly defined in the ICF and include all aspects of the physical (e.g., buildings, transportation system or the natural environment), social and attitudinal world (e.g., people’s attitudes, social support and relationships), as well as products (e.g., medication), and services, systems and policies (e.g., the availability of health care facilities). A recently published paper empirically corroborates the impact of EFs on severe disability by showing that social support, employment or being discriminated against because of health problems have a high impact on performance in several domains of functioning, defined in the ICF as how people actually conduct their everyday lives taking into consideration health conditions as well as the hindering or facilitating impact of EFs [3]. 

Comprehensive data and evidence about EFs are essential to identify potential intervention targets which may influence disability. This is emphasized in the United Nations Convention on the Rights of Persons with Disabilities (CRPD): “the importance of accessibility to the physical, social, economic and cultural environment, (…), in enabling persons with disabilities to fully enjoy all human rights and fundamental freedoms” (Preamble V) [4]. For all countries which have ratified the CRPD it is therefore essential to timely identify barriers and facilitators that have a high impact on disability as well as to understand to what extent and how environmental barriers hinder participation in society. 

Disability surveys are a direct source of information on disability and have the important task of providing not only disability rates but also of collecting comprehensive data on EFs. The framework of the ICF has become increasingly important for conceptualizing health and disability surveys since its endorsement in 2001, and is targeted in a considerable number of surveys performed since 2001 [5]. EFs are, however, still only partially covered across surveys [5], and as a consequence several surveys are not fully suitable to inform policy makers what aspects of the environment need to be influenced, so that persons with disability participate in society on an equal basis with others [5]. For instance, although discrimination due to health problems has an important impact on performance of persons with severe disability [3], attitudes of others were only covered by around half of the surveys included in a recent review of health and disability surveys [5]. 

One of the goals of the Model Disability Survey (MDS) project, initiated by the WHO and the World Bank (WB) in 2011, is to propose an instrument that will improve data collection on disability using the ICF as a framework [6]. The definition of disability proposed in the ICF is, however, complex and poses challenges in terms of data collection. In the MDS, WHO targets measuring disability in its complexity, and has therefore defined three components that must be measured and combined to achieve the goal of understanding disability as proposed in the ICF: capacity, performance and EFs. Performance is the term used to operationalize the outcome of the interaction of a health condition and EFs, *i.e.*, “how people live their lives” taking into account health conditions and several aspects of their environment. Capacity operationalizes, on the opposite, a heath state, *i.e.* the direct impact of a health condition on the body functions and the ability to carry out tasks and participate on society, not considering any impact from environmental factors. The current Alpha version of the MDS encompasses core modules to collect detailed information on (a) performance in several functioning domains taking into account health problems and EFs; (b) capacity—a measure of how health problems and the presence of health conditions directly impact how people function in multiple domains; and (c) EFs, broadly operationalized to include information on hindering aspects of the general environment, availability and need of personal assistance, assistive technologies and modifications, level of social support, attitudes of others, accessibility to information and regular use of medication. Moreover, being a general population survey, the MDS allows for the identification of EF barriers and needs of persons with mild, moderate and severe levels of difficulties in capacity. This is a prerequisite to design specific and detailed strategies to improve people’s lives and promote their full and equal participation in society as required in the CRPD. 

Taking advantage of the comprehensive information on EFs collected in the scope of a pilot implementation of the MDS in Cambodia, this paper introduces an approach for identifying the relative importance of EFs for persons with different levels of difficulties in capacity. The objective of the paper is to identify the EFs with the highest impact on the performance of persons experiencing different levels of difficulties in capacity. Three specific aims are addressed:

(1) to identify the EFs with the highest impact on performance in general,
(2) to identify the EFs with the highest impact on performance in persons with mild, moderate and severe levels of difficulties in capacity,
(3) to identify which EFs are the most relevant across all levels of difficulties in capacity.

## 2. Methods 

### 2.1. Study Design

In the implementation phase of the MDS the first cross-sectional pilot study was carried out using the Alpha version in the Cambodian provinces Kampong Thom and Kampot in August 2014. This pilot study targeted the feasibility and validity of the MDS in the cultural context of Cambodia. We used the data of this pilot study to identify the EFs which have the highest impact on performance in persons with mild, moderate and severe levels of difficulties in capacity by using random forest (RF) method.

### 2.2. Participants

The study population included a convenience sample of 500 adults aged 18 years or older who were interviewed in Khmer by trained interviewers of the National Institute of Statistics in Cambodia. Participants lived in the Kampong Thom and Kampot provinces and interviews were conducted in selected districts of these provinces to cover both urban and rural areas. A quota sample aligned to match the final survey population was used because it was more feasible to implement then a probability sample and was considered adequate for a pilot test. The convenience sample was selected following stratification by age, sex and education, and targeting the inclusion of healthy respondents as well as persons with impairments and health conditions. The convenience sample was therefore not representative of the future target population of the MDS, the general population, but included comparable proportions of persons without and with health conditions and impairments to test the feasibility and validity of the MDS in each of these groups. The study population and the MDS design have been described in detail elsewhere [6].

### 2.3. Variables

The individual questionnaire of the Alpha version of the MDS consists of seven sections. The present study uses data from sections “Environmental Factors” (section 3000), “Functioning” (section 4000) and “Health Conditions and Capacity” (section 5000). The MDS is available on request from ciezaa@who.int.

Performance—as the operationalization of disability and defined as how health states or capacity plays out in people’s lives in light of the environmental barriers or facilitators they encounter—was used as the dependent variable in this work. It was operationalized using a metrical performance scale ranging from 0, no problems in performance, to 100, extreme problems in performance. This metric scale was previously built using Polytomous Rasch analysis (Partial Credit Model (PCM) from Item Response Theory (IRT)) and questions from different domains of section 4000 [6].

Independent variables belonged to section 3000, EFs whose content is divided into seven parts: (1) Hindering or facilitating aspects of the general environment (nine questions); (2) Personal Assistance (five questions); (3) Assistive Devices (28 questions); (4) Support and Relationships (ten questions); (5) Attitudes of others (eleven questions); (6) Accessibility to Information (one question) and (7) Medication (one question). Questions used to address “Support and Relationships” encompassed the Oslo Social Support Scale [7]. With exception of questions on personal assistance and assistive devices, all questions used a 5-point Likert rating scale. Personal assistance questions target personal use and need of assistance and were combined into one single categorical variable with four possible attributes: person has but needs additional assistance, person has assistance and does not need additional assistance, person has no assistance but would need it, and person has no assistance and does not need any. Regarding assistive devices, only questions targeting the use of assistive devices were included. 

Age, sex, education and a capacity metric were handled as control variables and forced into the model, as disability associated differences were expected between the strata. Capacity was operationalized as a metric score ranging from 0 (no difficulties) to 100 (extreme difficulties). This metric was built in a previous study using Polytomous Rasch analysis and all capacity questions of section 5000 [6]. We controlled for capacity, *i.e.*, the ways health problems and the presence of health conditions affect how people function in multiple domains, because performance is understood as the outcome of the interaction between one’s level of intrinsic capacity and the built, social, political and attitudinal environment. This is in line with previous works targeting the impact of EFs on performance while controlling for capacity [8,9].

The Alpha version of the MDS can be obtained from the corresponding author upon request.

### 2.4. Statistical Methods

Descriptive statistics are used to characterize the sample and frequencies are used to describe the response patterns to EF questions. 

As there is no *a priori* assumption about the most relevant determinants (EFs) of performance and a large number of predictors, the random forest (RF) method was selected to identify the EFs which have the highest impact on performance. Random forest is a regression method used for ranking predictors and based on the random forest variable importance estimate [10]. To make a final prediction of the most relevant EFs, several individual regression trees are combined and all together build up a forest. The average of the predictions of all trees in the forest indicates the final prediction. In each tree conditional inference tests were used for each split to select the best split in an unbiased way. The predictor with the smallest *p*-value defines the best split (later version of RF according to Hothorn *et al.* [11]).

The applied RF algorithm contains one thousand individual regression trees which were combined to get a final ranking of importance of all environmental predictors included in the model with regard to their explanatory value for the dependent variable metric performance. Control variables (age, sex, education and capacity metric) were forced in the RF model. Each tree was fitted to a random sample of observations without replacement from the original sample [12]. The number of input variables randomly sampled as candidates at each node, which are called split variables in RF, was six (root of number of predictors). Each tree was fully developed (mincriterion was equal 0). The variable importance measure (VIM) is the average of the frequency with which the independent variables (EFs) appear in all thousand regression trees calculated to predict the dependent variable (metric performance) over all thousand trees. Therefore, we used the VIMs to get essentially unbiased rankings of the environmental predictors according to their association with the metric performance. The higher the VIM’s value the more relevant is the EF for the prediction of performance.

Vulnerable groups, *i.e.*, groups of persons with different levels of difficulties in capacity, are at risk of experiencing disability to different extents, and might face different EFs barriers and have different EFs needs. Random forest analyses were therefore carried out for the general study population and for three subpopulations stratified by vulnerability to disability due to different levels of difficulties in capacity—no and mild difficulties, moderate difficulties and severe difficulties—to infer specific lessons for the different subpopulations. Cut-off points for division into these subpopulations were previously set using the capacity metric and following the recommendations of the World report on disability (WRD) as well as the distribution of the capacity metric [6]. In brief, persons with capacity scores >47.4 were considered to have severe difficulties in capacity, whereas persons with capacity scores between 30 and 47.4 were considered to have moderate difficulties in capacity and finally people with capacity scores <30 were considered to have mild or no difficulties in capacity.

Multiple linear regression models were applied to determine how much of the variation in the performance metric can be explained by the independent variables with the highest VIMs. Independence of observations was assessed by a Durbin-Watson statistic and linear relationships were checked using scatterplots. Homoscedasticity (homogeneity of variance) was checked using a P-P diagram of standardized residuums. Multicollinearity was proof by checking the strength of correlations and the variance inflation factor (VIF). After model assumptions including collinearity and homoscedasticity were evaluated and the statistical significance was proven, variables were included stepwise (in descending order of importance) in a final single model according to the VIM’s ranking in the RF. The explained variance was calculated by using R^2^ and R^2^ adjusted. R^2^ adjusted was used as a reference to explore how much variance in performance was explained by the EFs with the greatest importance in RF. To decide how many EFs had an important impact on performance using R^2^ adjusted as a criterion, we defined the first decrease in R^2^ adjusted rounded to the third decimal place as a cut-off. Scree plots were used to visualize the decrease in VIMs and R^2^ (graphs not shown).

As there was a maximal amount for variable nonresponse of 1.2% (with one exception,,the question asking if workplace or school make it easy or hard to do desired things) missing values were imputed with the mean (metric variables) or median (ordinal variables) for calculating multiple linear regression models. The variable targeting if workplace or school make it easy or hard to do desired things had to be excluded from the analyses because many people in Cambodia do not have a regular job, and the variable had a high rate of structural missing values (not applicable). Data analysis was performed in R 3.1.2 (R Core Team, R Foundation for Statistical Computing, Vienna, Austria, 2014) and SPSS Statistics 23 (IBM Corp., Armonk, New York, NY, USA, 2015). For RF analyses the R function “cforest” was used (package “party”) [13].

## 3. Results 

Table 1 shows the characteristics of the study population. In the study population (*N* = 500) the mean of the metric performance score, used as dependent variable, was 40.4 (SD 16.1).

**Figure 1:**
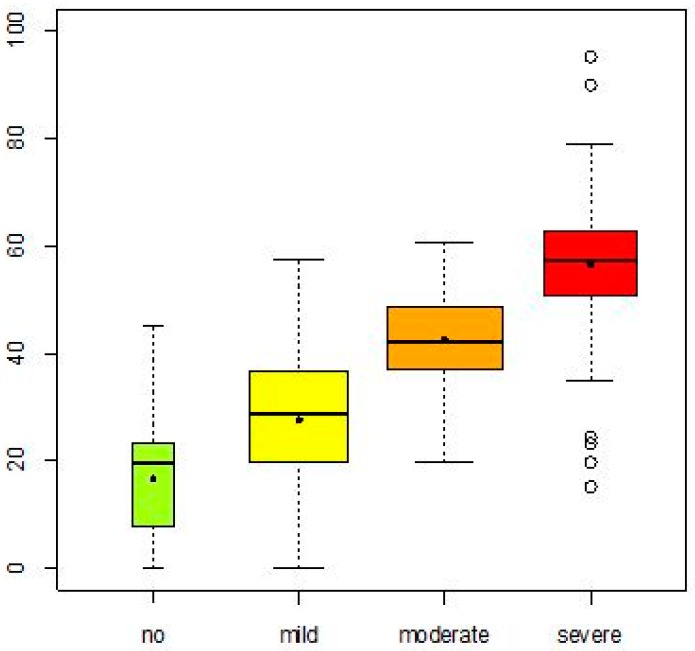
Boxplots showing the metric performance stratified by no, mild, moderate and severe level of difficulties in capacity. The y-axis represents the level of problems in performance in a metric scale raging from 0 (best performance) to 100 (worst performance); the x-axis represents the strata created based of severity of capacity difficulties.

Figure 1 presents the metric performance score of each of the strata—no, mild, moderate and severe level of difficulties in capacity—and demonstrates that participants with greater capacity difficulties also have greater problems in their performance. 

### 3.1. Environmental Factors with the Highest Impact on Performance

Considering the VIMs, altogether ten EFs had the highest impact on performance for the complete sample: six regarding hindering or facilitating aspects of places to socialize (14.3), the natural environment, e.g., temperature, climate (8.0), the transportation system (7.9), the dwelling (5.5), places to worship (5.2) and school or places to work (2.1). The remaining four EFs include: use of medication on regular basis (8.0); use and need of personal assistance (5.8); use of assistive devices for mobility and self-care (4.8) and the number of close relationships in the individual’s family (2.9). From this set four EFs showed striking high VIMs: hindering or facilitating aspects of places to socialize, the natural environment, the transportation system and use of medication.

The starting model including age, gender and capacity explained 68% of the variance in performance. The five EFs with the highest VIMs contributed most to the additional explained variance (74%). Adding further EFs to the model led to a small increase per EF in R^2^ adjusted (maximum 77%) (Table 2, column 3).

#### 3.1.1. Severe Levels of Difficulties in Capacity

Considering the VIMs, altogether twelve EFs had the highest impact on performance: four regarding hindering or facilitating aspects of places to socialize (10.1), places to worship (2.5), transportation system (2.5) and dwelling (1.9); two regarding relationships, namely number of close family members (11.6) and getting help from friends (2.8); two regarding attitudes, namely considering oneself a burden on society (4.1), a question adapted from the Attitudes to Disability Scale (ADS) [7], and participating in family decisions (2.5); two regarding assistive devices, namely the use of aids for mobility or self-care (4.1) and facilitators for participating in activities outside the home (1.4); and use of medication on a regular basis (3.7). From this set two EFs showed striking high VIMs: close relationships with family members and hindering or facilitating aspects of places to socialize.

The starting model including age, gender and capacity explained 48% of the variance in performance. The seven EFs with the highest VIMs contributed most to the additional explained variance (70%). Adding further EFs to the model led to a small increase per EF in R^2^ adjusted (maximum 73%) (Table 2, column 4). 

#### 3.1.2. Moderate Levels of Difficulties in Capacity

Considering the VIMs, altogether twelve EFs had the highest impact on performance: five regarding hindering or facilitating aspects of places to socialize (1.5), the transportation system (1.2), school or places to work (0.4), places such as shops and banks (0.4) and the natural environment, e.g., temperature, climate (0.4); three regarding relationships, namely the number of close neighbours (0.4) and friends (0.3) as well as the closeness of relationships to a partner (0.4); a question considering the use and need of personal assistance (1.2); the accessibility to information (0.7); use of medication on a regular basis (0.6) and a question regarding the feeling of being treated unfairly (0.3). 

The starting linear regression model including age, gender, education and capacity explained 25% of the variance in performance. The seven EFs with the highest VIMs contributed most to the additional explained variance (41%). Adding further EFs to the model led to a small increase per EF in R^2^ adjusted (maximum 47%) (Table 2, column 5). 

#### 3.1.3. Mild Levels of Difficulties in Capacity

Considering the VIMs, altogether fourteen EFs had the highest impact on performance: six regarding hindering or facilitating aspects of the natural environment, e.g., temperature, climate (2.7), lighting, noise or crowds (2.1), the dwelling (1.3), places to socialize (0.9), transportation system (0.8) and places such as shops and banks (0.6); five regarding attitudes, namely difficulties getting involved in society because of people’s attitudes (1.6), low expectations from people (1.5), considering oneself as a burden on society (0.9), living in dignity (0.8) and acceptance by other people (0.6); a question considering the use and need of personal assistance (1.0); use of medication on a regular basis (0.7) and the closeness of relationships to family members (0.6). The question considering hindering or facilitating aspects of the natural environment, e.g., temperature or climate had the highest absolute variable importance measurement (2.7). Using the VIMs as a reference, altogether three EFs had an important impact on performance. These include moreover aspects of the general environment (2.1), such as lighting, noise or crowds, and problems getting involved in society because of attitudes of other people (1.6). 

The stating model explained 27% of the variance. The ten EFs with the highest VIMs contributed most to the additional explained variance (43%). Adding further EFs to the model led to a neglectable increase per EF in R^2^ adjusted (maximum 44%) (Table 2, column 6).

### 3.2. Most Relevant Environmental Factors across All Levels of Difficulties in Capacity

Considering the fifteen most important VIMs based on results of RF analyses, Table 3 shows an overlap across all levels in hindering or facilitating aspects of places to socialize, the transportation system and the natural environment; in the use and need of personal assistance; and in the use of medication on a regular basis. The five EFs showing a complete overlap across all four groups are green-shaded.

## 4. Discussion

To our knowledge, this is one of the few studies identifying the EFs with the highest impact on performance—how people actually conduct their everyday lives taking into consideration health conditions as well as the hindering or facilitating impact of EFs—at the general population level. We included a convenience sample from persons with mild, moderate and severe levels of difficulties in capacity using data from a pilot study of the MDS in Cambodia. Our results show that hindering or facilitating aspects of places to socialize in community activities, transportation and the natural environment, as well as the use and need of personal assistance and the use of medication on a regular basis were the most important EFs across all levels of difficulties in capacity. EFs with the highest impact on performance differ, however, for the subgroups of persons with mild, moderate and severe difficulties in capacity pointing out that ranking EFs to identify priorities for policy and public health interventions must take into account to specify needs of these groups. 

Our results are in line with comparable studies targeting the impact of EFs on performance. A recently published study estimated the association between performance and EFs when controlling for capacity, using data from a national Spanish disability survey [3]. As this survey solely included people with severe disability, in terms of capacity, only findings for this group can be compared. Bostan *et al.* showed that social support, discrimination due to one’s health problems, work-related factors and the extent to which one’s health needs are addressed play especially an important role on performance. Social support might be concordant to the number of close family members and support of friends in our investigation. Also, discrimination due to one’s health problems could be consistent with our variable attitudes of others, such as considering oneself as a burden on society. As most of our respondents were not working, we excluded work-related factors from our analyses and cannot confirm the high impact of work-related factors on performance. Another comparable study considering a sample of people with musculoskeletal disorders and severe problems identified 13 EFs covering all aspects of the physical, social, attitudinal and political environment, which were significantly associated with performance when controlling for capacity [9]. We are in line with this study when considering the severe group regarding use and need of assistive devices, use of medication, hindering or facilitating aspects of the dwelling, personal assistance, relationships and attitudes of others. Even though an exact mapping of EFs is not possible because of quite different operationalizing of questions, our findings confirm that several aspects of EFs are needed for understanding performance. In addition, we have showed the importance of hindering or facilitating factors of places to socialize, worship, the dwelling, use and need of assistive devices for mobility and self-care, facilitators for participating in activities outside the home, use of medication on a regular basis and use and need of personal assistance for this group with severe difficulties in capacity.

Recently Prodinger *et al.* recommended a comprehensive set of ICF categories as a minimal standard for reporting and assessing functioning in clinical populations along the continuum of rehabilitation care—the ICF Rehabilitation Set [14]. If we compare the 12 EFs set proposed in the ICF Rehabilitation Set with the most important EFs for our strata with severe capacity difficulties, assuming these are the persons comparable to the sample used by Prodinger, we find agreement regarding hindering or facilitating aspects of places to socialize, places to worship, and of the own dwelling, social support of family and friends, use of assistive devices, use of facilitators for participating in activities outside the home and use of medication on a regular basis. The large overlap of EFs selected in both studies despite of different methodological designs and populations corroborates the robustness of our findings in terms of EFs that have a high impact on severe disability. 

Environmental factors overlapping across the strata with different levels of difficulties in capacity require particular attention on the part of policy makers or stakeholders in charge of public health interventions since they shed light on cross-cutting strategies and measures that can improve the lives of all persons with difficulties in capacity. We provided evidence that hindering or facilitating aspects of places to socialize in community activities, transportation and of the natural environment as well as the use and need of personal assistance and use of medication on a regular basis are EF with the highest impact on performance. These EFs point out that policies to improve participation in the community or the availability of personal assistance are of importance across levels of difficulties in capacity. It is essential to keep in mind, however, that we have used a convenience sample of a pilot study in Cambodia. The impact of transportation on performance, for instance, is highly important for everybody in Cambodia probably because the public transportation system is precarious in diverse parts of the country. This might be quite different in other countries. A universal identification of EFs valid across countries requires a population including samples from different countries and world regions. 

Although EFs overlapping across all levels of difficulties in capacity are important, our study points out that specific needs and barriers faced by persons experiencing different levels of difficulties in capacity must also be taken into account by policy makers or stakeholders in charge of public health interventions. We found that most of the important EFs targeting hindering or facilitating aspects are relevant in the group with mild levels of difficulties in capacity, worth mentioning are natural environment, lighting, noise or crowds, dwelling, places to socialize and transportation system. This might point to aspects of the country that affect the population in general as Cambodia is, inter alia, prone to extreme weather events and climate change [15]. When comparing all subsamples, the significance of relationships, such as the number of close family members or support of friends, became increasingly important with a higher level of difficulties in capacity. The importance of EFs targeting the attitudes of others was decreasing with a higher level of difficulties in capacity. Even though the negative effect on mental and physical health due to the experience of discrimination was confirmed, for instance, in a former meta-analysis [16], we showed that attitudes of others were palled by other EFs in persons experiencing high levels of difficulties in capacity. Use of medication on a regular basis is an important EF from moderate levels of difficulties in capacity upwards. Additionally, we demonstrated the high importance of the use and need of assistive devices for people with severe difficulties in capacity. Other investigations described the psychosocial benefits and a positive impact on the quality of life using assistive devices for severe disabilities, which is in line with our findings [17,18].

The CRPD, as it set out to promote and to ensure inclusion for persons experiencing disability on an equal basis with others, stresses the importance of environmental barriers that hinder people’s full and effective participation in society. Hindering or facilitating aspects of places to socialize were among the two most important EFs for severe and moderate difficulties in capacity as well as for the general sample regarding their impact on performance. As socializing and hence accessibility of social places are key aspects of human rights, our results empirically support the principles of the CRPD. Accordingly the significance of our findings, strengthen the mandate for actions applied to economic and social policies that focuses on availability and accessibility of social places. Research evidence has shown that there are successful opportunities to design buildings, facilities and cities targeting this universal human right, conscious of individual differences across disability groups [19]. The acknowledgment of how important inclusion is across all levels of disability is only possible in the present work because the MDS is designed as a general population survey. Bearing in mind that a universal social policy is desirable, a general population survey, like the MDS, is a suitable instrument to examine if people with different levels of disability as well as in comparison to people without disabilities benefit equally from participation opportunities.

Our study has some limitations. First, we used a convenience sample and consequently our results are not representative for the general population. Second, we analyzed data from the Cambodian MDS pilot study, and our findings should be therefore considered in the light of the specific political, economic and social context of Cambodia. Third, for subgroup analyses we obtained small sample sizes for each strata and people with no and mild difficulties in capacity must be combined into one group. Further studies with larger sample sizes considering separately persons without and with mild difficulties in capacity are necessary. Fourth, RF regression does not provide clear cut-off values, because the VIMs are used as a merely descriptive means of data. To overcome this limitation we estimated R^2^ adjusted when identifying the most relevant EFs referred to performance. Setting a cut-off for a selection of EFs with highest importance remained though challenging and somehow arbitrary. Additionally, ranking EFs does not account for their complex interaction among each other. Nevertheless, the ranking is still important in terms of providing policy makers with information on what could improve the everyday life of affected persons. The strength of our investigation is that we analyzed commonalities and differences in the importance of EFs regarding performance for the general sample and for people with different levels of difficulties in capacity. 

## 5. Conclusions 

In an effort to identify the EFs with the highest impact on performance of persons with different levels of difficulties in capacity, our results showed that hindering or facilitating aspects of places to socialize in community activities, transportation and the natural environment as well as the use and need of personal assistance and the use of medication on a regular basis were the most important EFs across all strata. However, the EFs with the highest impact on performance were different for persons with mild, moderate and severe difficulties in capacity, pointing out the different needs of each of these vulnerable groups. The RF regression method to show which EFs have the highest impact on performance of people with different levels of difficulties in capacity has been applied for the first time. It was shown to be an appropriate method, suitable for application along the path to rank the importance of EFs, including health and disability surveys, and in doing so to identify barriers, needs, and priorities while taking into account the level of difficulties in capacity experienced. 

## Figures and Tables

**Table 1:** Sociodemographic characteristics of the study population (*N* = 500).

Sociodemographic Characteristics	Level	Severe Difficulties in Capacity		Moderate Difficulties in Capacity		Mild Difficulties in Capacity		Complete Sample ^a^	
		*N*	%	*N*	%	*N*	%	*N*	%
**Sample size**		128	25.6	200	40.0	171	34.2	500	100.0
**Gender**	Male	52	40.6	69	34.5	72	42.1	193	38.6
	Female	76	59.4	131	65.5	99	57.9	307	61.4
**Education**	No schooling or never completed any grade	41	32.0	34	17.0	20	11.7	95	19.0
	Elementary education	54	42.2	96	48.0	63	36.8	214	42.8
	Secondary school	30	23.4	62	31.0	78	45.6	170	34.0
	Other	3	2.3	8	4.0	10	5.8	21	4.2
		**Mean**	**SD**	**Mean**	**SD**	**Mean**	**SD**	**Mean**	**SD**
**Age, years**		51.1	15.8	41.7	12.5	37.7	11.6	42.8	14.2
**Metric capacity score ^b^**		56.8	9.1	38.7	5.0	14.0	10.7	34.9	18.7
**Metric performance score ^c^**		56.4	12.6	42.6	7.7	25.9	12.5	40.4	16.1

**^a^** One participant could not be allocated to any strata, because a calculation of the metric capacity score was not possible. Nevertheless, this person was taken into consideration for analyses, therefore *N* = 500; *N* = number; SD = standard deviation; **^b^** Capacity metric: Value range is from 0 to 100, meaning the higher the score the greater the difficulties experienced because of health related decrements in functioning domains; **^c^** Metric performance score: Value range is from 0 to 100, meaning the higher the score the greater the problems experienced in daily life.

**Table 2:** Results of the random forest (RF) and multiple linear regression models to determine the most important environmental factors (EFs) with the highest impact on performance.

Category	Variable	Complete Sample *N* = 500				Severe Difficulties in Capacity *N* = 128				Moderate Difficulties in Capacity *N* = 200				Mild Difficulties in Capacity *N* = 171			
		All Variables				All Variables				All Variables				All Variables			
		VIM ^a^	Rank	R²	R²adj ^b^	VIM ^a^	Rank	R²	R²adj ^b^	VIM ^a^	Rank	R²	R²adj ^b^	VIM ^a^	Rank	R²	R²adj ^b^
**Control ^c^**	Age	11.4		0.7	0.681	9.6		0.5	0.483	0.3		0.3	0.253	−0.2		0.3	0.272
**Control**	Sex	0.2				−0.1				0.4				0.7			
**Control**	Level of education	2.5				0.7				0.1				3.3			
**Control**	Capacity metric score	94.4				34.2				7.7				30.6			
**General EF ^d^**	Work/school	**2.1**	10	0.8	0.742	0.4	20	0.9	0.718	**0.4**	6	0.4	**0.373**	0.1	26	0.7	0.430
**General EF**	Health facilities	1.1	16	0.8	0.761	0.7	15	0.8	0.720	0.2	16	0.6	0.432	−0.1	32		
**General EF**	Places to socialize	**14.3**	1	0.7	**0.712**	**10.1**	2	0.7	**0.621**	**1.5**	1	0.3	**0.316**	**0.9**	7	0.5	**0.409**
**General EF**	Shops banks	1.7	12	0.8	0.741	0.1	26	0.9	0.686	**0.4**	8	0.5	0.400	**0.6**	14	0.6	0.430
**General EF**	Worship	**5.2**	7	0.7	0.736	**2.5**	8	0.8	0.702	0.1	21	0.6	0.415	0.0	27	0.7	0.421
**General EF**	Transportation	**7.9**	4	0.7	**0.728**	**2.5**	10	0.8	0.698	**1.2**	3	0.4	**0.347**	**0.8**	9	0.5	**0.421**
**General EF**	Dwelling	**5.5**	6	0.7	0.737	**1.9**	11	0.8	0.705	0.1	19	0.6	0.427	**1.3**	5	0.5	**0.384**
**General EF**	Natural environment	**8.0**	2	0.7	**0.716**	0.9	14	0.8	0.709	**0.4**	9	0.5	0.404	**2.7**	1	0.3	**0.291**
**General EF**	Lighting noise crowds	1.7	11	0.8	0.741	0.3	21	0.9	0.718	0.0	30			**2.1**	2	0.4	**0.318**
**Personal Assistance**	Assistance summary	**5.8**	5	0.7	**0.738**	**3.1**	6	0.7	**0.691**	**1.2**	2	0.4	**0.337**	**1.0**	6	0.5	**0.388**
**Assistive Devices**	Mobility and self-care	**4.8**	8	0.8	0.736	**4.1**	4	0.7	**0.659**	0.0	28	0.7	0.462	0.0	28	0.7	0.416
**Assistive Devices**	Seeing	0.4	26	0.8	0.767	0.1	28	0.9	0.669	0.0	24	0.6	0.421	0.0	29	0.7	0.414
**Assistive Devices**	At home	1.4	14	0.8	0.753	0.5	18	0.8	0.731	0.2	18	0.6	0.435	0.0	30	0.7	0.407
**Assistive Devices**	In the community	0.2	32	0.8	0.768	**1.4**	12	0.8	0.702	0.3	13	0.5	0.391	−0.2	35		
**Relationships**	Help family member	0.1	36	0.8	0.768	0.2	25	0.9	0.691	0.0	32			−0.2	34		
**Relationships**	Help friends	0.7	21	0.8	0.764	**2.8**	7	0.8	**0.703**	0.0	27	0.7	0.466	−0.2	36		
**Relationships**	Help neighbours	0.4	27	0.8	0.767	0.0	29	0.9	0.646	0.1	20	0.6	0.419	0.4	15	0.6	0.433
**Relationships**	Close partner	0.2	33	0.8	0.767	−0.1	34			**0.4**	10	0.5	0.407	0.2	22	0.7	0.407
**Relationships**	Close family	0.4	25	0.8	0.767	0.3	22	0.9	0.713	0.0	33			**0.6**	13	0.6	0.444
**Relationships**	Close friend	0.5	23	0.8	0.765	0.7	17	0.8	0.733	0.2	17	0.6	0.427	0.2	20	0.7	0.430
**Relationships**	Close neighbour	0.2	34	0.8	0.767	0.0	31			0.3	14	0.6	0.441	0.3	17	0.6	0.430
**Relationships**	Number close family	**2.9**	9	0.8	0.740	**11.6**	1	0.6	**0.579**	0.3	15	0.6	0.440	0.2	23	0.7	0.400
**Relationships**	Number close friends	0.3	30	0.8	0.768	0.2	24	0.9	0.696	**0.3**	12	0.5	0.395	0.1	24	0.7	0.394
**Relationships**	Number close neighbour	0.2	35	0.8	0.769	−0.1	35			**0.4**	7	0.5	**0.408**	0.0	31		
**Attitudes of others**	Participate family decisions	0.3	29	0.8	0.768	**2.5**	9	0.8	0.705	0.0	29	0.7	0.470	−0.2	37		
**Attitudes of others**	Society involvement	0.8	20	0.8	0.764	0.3	23	0.9	0.701	0.0	34			**1.6**	3	0.4	**0.339**
**Attitudes of others**	Treat unfair	0.4	28	0.8	0.768	0.0	32			**0.3**	11	0.5	0.399	0.3	18	0.6	0.431
**Attitudes of others**	Own choices	0.5	24	0.8	0.766	1.0	13	0.8	0.712	0.0	26	0.7	0.461	0.1	25	0.7	0.383
**Attitudes of others**	Big decisions	1.1	17	0.8	0.763	0.1	27	0.9	0.677	−0.1	36			0.3	19	0.7	0.434
**Attitudes of others**	Accept you	0.3	31	0.8	0.767	0.0	30	0.9	0.669	0.1	22	0.6	0.414	**0.6**	12	0.6	0.428
**Attitudes of others**	Respect you	0.0	37	0.8	0.768	0.4	19	0.9	0.726	0.0	25	0.6	0.410	−0.2	33		
**Attitudes of others**	Burden on society	1.4	15	0.8	0.760	**4.9**	3	0.7	**0.659**	0.0	31			**0.9**	8	0.5	**0.418**
**Attitudes of others**	Impatient with you	0.8	18	0.8	0.763	−0.1	36			−0.1	37			0.4	16	0.6	0.431
**Attitudes of others**	Expectations from you	1.5	13	0.8	0.753	−0.1	37			0.1	23	0.6	0.424	**1.5**	4	0.4	**0.374**
**Attitudes of others**	Living in dignity	0.8	19	0.8	0.763	0.7	16	0.8	0.733	0.0	35			**0.8**	10	0.6	**0.425**
**Accessibility to information**	Access information	0.5	22	0.8	0.764	−0.1	33			**0.7**	4	0.4	**0.362**	0.2	21	0.7	0.422
**Medication**	Medication regularly	**8.0**	3	0.7	**0.725**	**3.7**	5	0.7	**0.678**	**0.6**	5	0.4	**0.372**	**0.7**	11	0.6	0.422
	**Correlation Observed-Predicted**	**0.9**				**0.8**				**0.8**				**0.8**			

**^a^** VIM: Variable importance measures estimated with random forest regression; **^b^** R² adj: R² adjusted showing the increase in explained variance calculated with classical multiple linear regression analyses by adding the determinants stepwise in descending rank order into the model; **^c^** Control: All models were controlled for age, gender, level of education and capacity; **^d^** EF: Environmental Factors. Note: Metric performance, a single metric score estimating the impact of performance, was used as dependent variable for all analyses. The most important EFs for each level of capacity as well as for the general sample are marked in bold. The green color scaling shows the rank order of the EFs (the darker the green color, the more important the EF).

**Table 3:** Most relevant environmental factors (EFs) across all levels of disability and the complete sample.

Category	Variable	Complete Sample	Severe Difficulties in Capacity	Moderate Difficulties in Capacity	Mild Difficulties in Capacity
**General EF**	Work school	x		x	
**General EF**	Health facilities		x		
**General EF**	Places to socialize	x	x	x	x
**General EF**	Shops banks	x		x	x
**General EF**	Worship	x	x		
**General EF**	Transportation	x	x	x	x
**General EF**	Dwelling	x	x		x
**General EF**	Natural environment	x	x	x	x
**General EF**	Lighting noise crowds	x			x
**Personal Assistance**	Assistance summary	x	x	x	x
**Assistive Devices**	Mobility and self-care	x	x		
**Assistive Devices**	Seeing				
**Assistive Devices**	At home	x			
**Assistive Devices**	In the community		x	x	
**Relationships**	Help family member				
**Relationships**	Help friends		x		
**Relationships**	Help neighbours				x
**Relationships**	Close partner			x	
**Relationships**	Close family				x
**Relationships**	Close friend				
**Relationships**	Close neighbour			x	
**Relationships**	Number close family	x	x	x	
**Relationships**	Number close friends			x	
**Relationships**	Number close neighbour			x	
**Attitudes of others**	Participate family decisions		x		
**Attitudes of others**	Attitudes society involvement				x
**Attitudes of others**	Treat unfair			x	
**Attitudes of others**	Own choices		x		
**Attitudes of others**	Big decisions				
**Attitudes of others**	Accept you				x
**Attitudes of others**	Respect you				
**Attitudes of others**	Burden on society	x	x		x
**Attitudes of others**	Impatient with you				
**Attitudes of others**	Expectations from you	x			x
**Attitudes of others**	Living in dignity				x
**Accessibility to information**	Access information			x	
**Medication**	Medication regularly	x	x	x	x

x: Variable was among the top fifteen important ranks based on the results of random forest regression analyses; EF: Environmental Factors; Green-shaded fields: Complete overlapping across all four groups. Grey-shaded fields: Absolutely no overlapping across all four groups.
